# Taxonomic Reliability of DNA Sequences in Public Sequence Databases: A Fungal Perspective

**DOI:** 10.1371/journal.pone.0000059

**Published:** 2006-12-20

**Authors:** R. Henrik Nilsson, Martin Ryberg, Erik Kristiansson, Kessy Abarenkov, Karl-Henrik Larsson, Urmas Kõljalg

**Affiliations:** 1 Department of Plant and Environmental Sciences, Göteborg University Göteborg, Sweden; 2 Department of Mathematical Statistics, Chalmers University of Technology Göteborg, Sweden; 3 Institute of Botany and Ecology, University of Tartu Tartu, Estonia; Pasteur Institute, France

## Abstract

**Background:**

DNA sequences are increasingly seen as one of the primary information sources for species identification in many organism groups. Such approaches, popularly known as barcoding, are underpinned by the assumption that the reference databases used for comparison are sufficiently complete and feature correctly and informatively annotated entries.

**Methodology/Principal Findings:**

The present study uses a large set of fungal DNA sequences from the inclusive International Nucleotide Sequence Database to show that the taxon sampling of fungi is far from complete, that about 20% of the entries may be incorrectly identified to species level, and that the majority of entries lack descriptive and up-to-date annotations.

**Conclusions:**

The problems with taxonomic reliability and insufficient annotations in public DNA repositories form a tangible obstacle to sequence-based species identification, and it is manifest that the greatest challenges to biological barcoding will be of taxonomical, rather than technical, nature.

## Introduction

Species identification relies heavily on DNA sequence comparison in many groups of organisms, particularly those in which distinguishing morphological characteristics come thinly seeded. Such processes, increasingly known as *barcoding*, hold great promise for simplifying and standardizing the identification of biological specimens [1]–[2]. The course of action is straightforward: some predefined DNA region of the organism is sequenced and compared for similarity in an inclusive database for sequence data such as the longstanding International Nucleotide Sequence Database [3] (INSD: GenBank, EMBL, and DDBJ) which is the most widely used sequence repository in the field. The result is used in the taxonomic annotation of the new sequence, which usually is submitted to the database under the inferred name. Such a procedure leans on three central but, interestingly, somewhat implicit assumptions [4]–[5]:

1. that the reference database features a satisfactory taxonomic sampling of sequences
2. that the sequences in the reference database are correctly identified and annotated
3. that the process of translating the comparison into species names is standardized, universally adopted, and not easily misunderstood

In the case of fungi, none of these criteria are met to any satisfactory extent:

1. Less than 1% of the estimated 1.5 million extant species of fungi have been sequenced for the ITS region, the most widely used locus for species identification in the fungi [6]–[7].
2. It has been suggested that a considerable portion-perhaps as much as 20%-of all fungal sequences deposited in INSD may be incorrectly annotated to species level [8], though rigorous statistics are lacking.
3. Newly generated sequences are typically identified using DNA-similarity searches like BLAST [9]. These are bound by criteria 1 and 2 and are associated with a range of additional complications such that their use for taxonomic identification has been cautioned in recent years [5], [10]–[11].

One does not have to stretch ones imagination to see how unfortunate decisions and circumstances, once effectuated, will not only remain in but also propagate through the various public sequence repositories through subsequent searches and submissions. Indeed, contemporary scientific literature is strewn with cases of mistaken species identities resulting from compromised DNA sequence comparison [10], [12]–[13].

But exactly how much reliance could be placed on the taxonomic annotations of publicly available sequences–how large a proportion of these are disputable? The present study aims to generalize previous sectional estimates by *in silico* analysis of a large set of fungal DNA sequences from INSD for various statistics. On the basis of the odd 51,000 fungal ITS sequences currently available, we carried out serial sequence similarity analysis and, for a subset of the sequences, external comparison to present objective statistics on the taxonomic reliability of fungal ITS sequences in INSD. Fungi form a large and ubiquitous group of organisms where species identification on morphological grounds often falls short and where the use of DNA sequence analysis for eukaryote species identification was once pioneered [14]. They therefore constitute an appealing model group for estimation of taxonomic reliability in public sequence databases under authentic circumstances.

## Materials and Methods

The ITS region is a multi-copy, transcribed but non-coding and easily amplified region of the ribosomal DNA [15]–[16]. It has become the standard locus for species identification–often even delimitation-in the fungi due to its high variability [17]–[18]. A local copy of all 51354 INSD fungal ITS sequences was created and kept up-to-date through weekly synchronization (Supporting Information). To nuance the representation of fungal diversity, the sequences were divided into those *fully identified* (identified to species level) and those *insufficiently identified* (not identified to species level) using regular expressions on the INSD organism specification field [7]. All sequences were compared against each other for similarity using NCBI-BLAST and the results were analyzed for various statistics (Table 1). As a second reference point, for the cases where the fungal taxonomic reference database UNITE [19] featured fully identified but independent ITS sequences from species also represented in the INSD dataset, the UNITE sequences were run against the latter to estimate ones chances of obtaining the correct name as the topmost BLAST match in INSD. For purposes of sequence comparison with BLAST, a *thorough match* was conservatively defined (Supporting Information) as to be far more stringent than the informal 3% rule of sequence dissimilarity sometimes evoked for species delimitation among bacteria and other organisms [20]–[21]. Sequences in match-pairs that satisfy the thorough match-criteria are referred to as *applicable*.

**Table 1:**
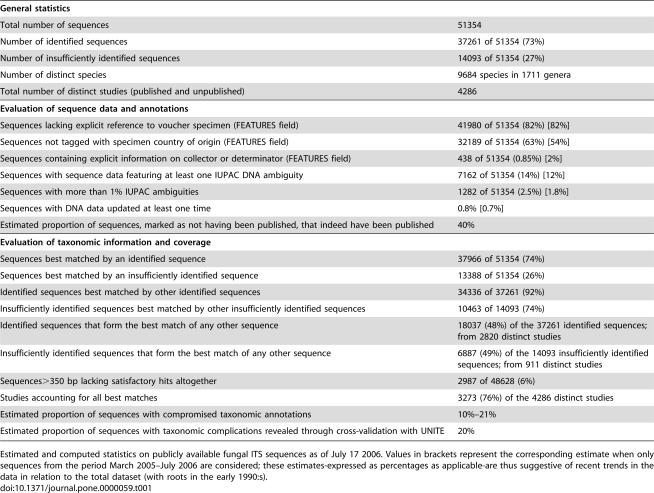
A fungal perspective on data reliability in INSD.

General statistics	
Total number of sequences	51354
Number of identified sequences	37261 of 51354 (73%)
Number of insufficiently identified sequences	14093 of 51354 (27%)
Number of distinct species	9684 species in 1711 genera
Total number of distinct studies (published and unpublished)	4286
**Evaluation of sequence data and annotations**	
Sequences lacking explicit reference to voucher specimen (FEATURES field)	41980 of 51354 (82%) [82%]
Sequences not tagged with specimen country of origin (FEATURES field)	32189 of 51354 (63%) [54%]
Sequences containing explicit information on collector or determinator (FEATURES field)	438 of 51354 (0.85%) [2%]
Sequences with sequence data featuring at least one IUPAC DNA ambiguity	7162 of 51354 (14%) [12%]
Sequences with more than 1% IUPAC ambiguities	1282 of 51354 (2.5%) [1.8%]
Sequences with DNA data updated at least one time	0.8% [0.7%]
Estimated proportion of sequences, marked as not having been published, that indeed have been published	40%
**Evaluation of taxonomic information and coverage**	
Sequences best matched by an identified sequence	37966 of 51354 (74%)
Sequences best matched by an insufficiently identified sequence	13388 of 51354 (26%)
Identified sequences best matched by other identified sequences	34336 of 37261 (92%)
Insufficiently identified sequences best matched by other insufficiently identified sequences	10463 of 14093 (74%)
Identified sequences that form the best match of any other sequence	18037 (48%) of the 37261 identified sequences; from 2820 distinct studies
Insufficiently identified sequences that form the best match of any other sequence	6887 (49%) of the 14093 insufficiently identified sequences; from 911 distinct studies
Sequences>350 bp lacking satisfactory hits altogether	2987 of 48628 (6%)
Studies accounting for all best matches	3273 (76%) of the 4286 distinct studies
Estimated proportion of sequences with compromised taxonomic annotations	10%–21%
Estimated proportion of sequences with taxonomic complications revealed through cross-validation with UNITE	20%

Estimated and computed statistics on publicly available fungal ITS sequences as of July 17 2006. Values in brackets represent the corresponding estimate when only sequences from the period March 2005–July 2006 are considered; these estimates-expressed as percentages as applicable-are thus suggestive of recent trends in the data in relation to the total dataset (with roots in the early 1990:s).

## Results and Discussion

### Proportion of compromised taxonomic annotations

The results are summarized in Table 1, which portrays a variegated picture of the taxonomic status of publicly indexed fungal sequences. Based on the conservative criteria defined for a thorough BLAST match and the discriminative variability of the ITS region, one would expect any such thoroughly matching pair to be conspecific. Yet 11% of all 15491 applicable sequences find thorough matches in other congeneric but heterospecific sequences, and another 7% among species of a different genus. When synonyms are accounted for, these correspond to 3231 distinct accession numbers such that a minimum of 10% and a maximum of 21% of the applicable sequences have compromised taxonomic annotations (Supporting Information). These entries form, in turn, the best matches of 5% of all insufficiently identified sequences, such that in a worst-case scenario, one in every twenty insufficiently identified sequences finds its most similar counterpart among entries whose taxonomic annotation can be questioned.

That 10–21% of the INSD sequences have incorrect or unsatisfactory taxonomic annotations translates into a matter of concern for the researcher seeking to establish the taxonomic affiliation of newly generated sequences. To obtain a clearer picture of the extent to which this process will be hampered by the compromised entries, the sequence identification procedure was reproduced through the use of UNITE, a highly filtered, closed-submission taxonomic database for reliable ITS-based identification of mycorrhizal fungi (http://unite.ut.ee). We employed the 240 species present in both INSD and the UNITE databases such that the UNITE sequences were used as input for comparison in INSD (Supporting Information). As the taxonomic affiliations of the UNITE sequences are well-known and -documented, the proportion of times a different taxonomic affiliation is suggested by INSD-even though a conspecific ITS sequence is present therein-represents a rational estimate of the impact of taxonomically compromised annotations in INSD. We found that one has on average a 20% (49/240) chance of obtaining a different species name on top of the INSD BLAST hit list, each such case hinting at a compromised annotation of either the topmost match or the purportedly conspecific INSD sequence (or even both). In a further 8% (20/240) of the cases, the correct species name was present in the topmost region of the hit list but was obscured by the presence of insufficiently identified sequences, such that one would be reluctant to annotate ones sequence after the best fully identified match. Jointly these estimates imply that the taxonomic and nomenclatural problems in public sequence databases are more far-reaching than previously assumed and that this has considerable repercussion on sequence-based species identification.

### Insufficiently identified sequences, orphans, and other compounding factors

More than 27% of all fungal ITS sequences in INSD are insufficiently identified, and the majority (74%) of these find their best match in other insufficiently identified-rather than fully identified-sequences. Similarly, over 90% of the fully identified sequences find their best matches in other fully identified sequences. In other words, the two sequence classes constitute two largely separate entities, both of which convey information not present in the other.

Six percent of all sequences over 350 bp lack good BLAST matches altogether (i.e., have an E-value of >0.0 as reported by BLAST). These outliers probably represent a mix of species whose closest relatives have not been sequenced and species that lack close, extant relatives. Two thirds of these sequences are fully identified; the oldest sequence with an unsatisfactory BLAST match has resided in INSD for a full 14 years. Interestingly, 85% of the fully identified sequences that fail to find a thorough match do so in the presence of other purportedly congeneric sequences, and 35% even in the presence of other purportedly conspecific sequences.

The observation that a comparatively small set of sequences explains a disproportionally large part of the results (Table 1) is probably best viewed as an indication of a highly patchy and non-random taxonomic distribution of species sampled. Roughly half of both the identified and the insufficiently identified sequences do not constitute the best BLAST match of any other sequence. Similarly, 76% of all mycological studies account for 100% of all best BLAST matches, such that there are over 1000 studies in INSD whose sequences do not constitute the best match of any other sequence (a *study* is defined as a distinct combination of the INSD AUTHORS and TITLE fields as to correspond to a published or unpublished scientific manuscript). A full 55% of all sequences are best matched by another sequence from the same study.

Sequence annotations play an important role for the researcher trying to verify alleged names and taxonomic integrities. However, many entries in INSD prove to be both devoid of vital information and outdated (Table 1). For example, 82% of the sequences lack explicit reference to a voucher specimen, 63% are not tagged with specimen country of origin, and 42% of all sequences are marked as not having been published in spite of the fact that about 40% of these indeed have been (Supporting Information). Although 14% of all sequences contain DNA ambiguities, less than 1% of all sequences have ever been updated. That these issues pose a further obstacle to sequence identification needs little iteration.

### Primary data - a challenge for biological barcoding

The present study suggests that the taxonomic reliability in public databases is not satisfactory, and that the problem shows little tendency for self-amelioration over time (Table 1). This is worrisome, particularly since DNA sequences have been opined as the primary information source in barcoding-type approaches to species identification (where reference DNA sequences serve as arbiters-barcodes-of conspecificity). It is apparent from Table 1 that the major sequence databases are not optimally suited to serve as barcoding engines as they presently stand; new techniques and strategies for data indexation and verification will have to be explored to address the above shortcomings [5], [22]. It is, however, not in technology that the greatest challenge to barcoding lies; rather, it is in the integrity of the primary data itself [23]–[25] (Table 1). As the results presented herein suggest, the relation of species and species names-taxonomy - to barcoding could be only one: that of the *primus motor*. No technical feats could ever make up for compromised primary data or lack of such data altogether.

The large body of insufficiently identified fungi in INSD constitutes a silent plea for a wide and generalized sequencing effort of well-identified and -annotated [type] specimens residing in herbaria worldwide to form the basis for such barcoding initiatives. This will without doubt be a painstaking undertaking involving taxonomic experts in all groups of fungi. The approach taken by the UNITE database has been to cover as many genera of fungi as possible at the temporary expense of intrageneric completeness. That approach finds support in the present study: in order to avoid the current situation where insufficiently identified sequences amass and obscure similarity searches in the public sequence databases, select reference sequences covering the whole range of fungal diversity need be made available as early on as possible.

### Conclusions

The species is in many ways the basic unit in biology, and the ever-increasing rate at which DNA sequences are released and used for scientific research prompts us to make any effort to verify that these are tagged with correct names. Sadly, more than 10% of all publicly available fungal ITS sequences have compromised taxonomic annotations, and the information needed to evaluate whether any given name is reasonable is in many cases simply not there. The inherent difficulty in species identification in the fungi, however, suggests that these estimates need not necessarily reflect the status of the total body of DNA sequences. Even so, caution and patience should be attributes of anyone seeking to identify species through DNA sequence data alone.

Barcoding-type approaches will doubtlessly be a central and most valuable element in future species identification, though contemporary major sequence repositories are not optimally suited for such operation. While we can expect technological advancements to eliminate many of the problems faced at present, the taxonomical aspect of the DNA sequences remains a substantial concern. Taxonomy lays at the heart of sequence-mediated species identification, and unlike the latter it forms a poor candidate for automation. Sadly the declining number of taxonomists is a problem for which no shortcuts exist and moreover one whose immediate resolution does not seem to be looming on the horizon.

## Supporting Information

Technical Information Detailed technical description of estimates used in the manuscript
(0.10 MB PDF)
