# Supplementary material for: Taxonomic Reliability of DNA Sequences in Public Sequence Databases: A Fungal Perspective
Source: PLoS One. 2006 Dec 20;1(1):e59. doi: 10.1371/journal.pone.0000059 (PMC1762357; doi:10.1371/journal.pone.0000059)
Supplement: Technical Information — Detailed technical description of estimates used in the manuscript (0.10 MB PDF) [file pone.0000059.s001.pdf]

# Supplementary Information

*"A local copy of all 51354 INSD fungal ITS sequences was created and kept up-to-date through weekly synchronization (Supplementary Methods)."*

INSD was queried on remote using BioPerl (`http://www.bioperl.org`) running on a Mandriva Linux 10.2 machine (`http://www.mandriva.com`). The INSD query string used was `'(("Fungi"[Organism] AND (200[SLEN] : 3000[SLEN])) AND (((ITS1[titl] OR ITS2[titl]) OR 5.8S[titl]) OR "internal transcribed spacer"[titl] OR "internal transcribed spacers"[titl]))'`. It excludes some few very short and very long sequences as well as sequences with deviant annotations and fungal sequences marked as non-fungal. The query string matches at least 98 % of all fungal ITS sequences available. All files pertaining to this study are available at `http://andromeda.botany.gu.se/ographis.html`

*"For purposes of sequence comparison with BLAST, a thorough match was conservatively defined (Supplementary Methods) as to be far more stringent than the informal 3% rule of sequence dissimilarity sometimes evoked for species delimitation among bacteria and other organisms[20, 21]"*

A "*thorough match*" was defined as a fully identified sequence with a length of between 350 and 650 basepairs (bp) that finds a fully identified sequence as its best (topmost) BLAST match such that the matching region covers - and is identical with - at least 98.5% of the shortest of the two sequences; these arbitrary values were chosen so as to go well with inter- and intraspecific variation in ITS sequences and were verified for efficacy against several published datasets. The length restrictions serve to rule out exceedingly long sequences from the comparison (typically those featuring a large, additional part of the contiguous nLSU gene) to make certain that it really is the variable ITS region that forms the basis for the comparison.

*"When synonyms are accounted for, these correspond tot 3231 distinct accession numbers such that a minimum of 10% and a maximum of 21% of the applicable sequences have compromised taxonomic annotations (Supplementary Information)."*

We thus have a list of pairs of fully identified, appropriately long, and extremely well-matching sequences. The left column of this list contains 2531

accession numbers that are all distinct; the right column holds their thoroughly best BLAST matches; and though each pair is exceedingly similar sequence-wise, they are lexicographically heterospecific such that at least one sequence in each pair can be hypothesized to convey a compromised taxonomic annotation [synonyms were located and removed prior to these steps]. One and the same accession number can be present more than once in the list (since any given sequence can form the best BLAST match of any number of sequences; right column). There are 3231 distinct accession numbers in the list, and the worst-case scenario is that they all (3231 / 15491 = 21 %) are incorrectly annotated taxonomically. Since the left column contains only distinct accession numbers (2531 / 2531) whereas the right column does not (1471 / 2531), the best-case scenario is that only all the accession numbers in the right column are incorrectly annotated to species level (1471 / 15491 = 10 %). The true value is hence found in the interval 10 - 21 %. There is no reason to think that the applicable sequences are not representative for the INSD data as a whole.

Prior to these calculations, all pairs were compared for synonymy and anamorph - teleomorph associations in Index Fungorum (`http://www.indexfungorum.org`) and the CBS Anamorph-Teleomorph database (`http://www.cbs.knaw.nl/databases/`). Conspecificity was indicated for 11 % of the initial 2862 pairs, with potential or hypothetical conspecificity indicated for an additional 6 %. Those 11% for which synonymy was clearly indicated were removed from subsequent comparisons; those 6 % whose synonymy was uncertain or questionable were left in. Data as of July 17 2006.

*"We employed the 240 species present in both INSD and the UNITE databases such that the UNITE sequences were used as input for comparison in INSD (Supplementary Information)."*

All species represented by ITS sequences of sufficient length (≥400 bp) in INSD and UNITE were selected. 240 of these were found to represent distinct specimens (i.e., the annotation-wise conspecific sequences were found to come from distinct vouchers / collections). The UNITE sequences - for which the species names were known and well-documented - were fed sequentially to NCBI-BLAST at INSD. We kept track of the proportion of times a different species name was suggested by the BLAST search, as well as the proportion of times that the correct name was present in the topmost region of the BLAST hit list but was obscured - superseded -

by insufficiently identified sequences. Synonyms were accounted for. Data as of May 2006.

*"For example, 82 % of the sequences lack explicit reference to a voucher specimen, 63 % are not tagged with country of origin, and 42 % of all sequences are marked as not having been published in spite of the fact that about 40% of these indeed have been (Supplementary Information)."*

One hundred distinct studies marked as "Unpublished" were randomly selected from the dataset. Joint search with the author and title fields of the studies were then made in ISI Web of Science® and Google Scholar (`http://scholar.google.com`) to see whether the studies in fact have been published. The detailed results are available at `http://andromeda.botany.gu.se/ographis.html`
